# *Anopheles gambiae* (*s.l.*) is found where few are looking: assessing mosquito diversity and density outside inhabited areas using diverse sampling methods

**DOI:** 10.1186/s13071-020-04403-9

**Published:** 2020-10-15

**Authors:** Patric Stephane Epopa, Abdoul Azize Millogo, Catherine Matilda Collins, Ace R. North, Mark Quentin Benedict, Frederic Tripet, Samantha OʼLoughlin, Roch K. Dabiré, Georges Anicet Ouédraogo, Abdoulaye Diabaté

**Affiliations:** 1 Institut de Recherche en Sciences de la Santé/Centre Muraz, Bobo-Dioulasso, Burkina Faso; 2 Institut des Sciences des Sociétés, Ouagadougou, Burkina Faso; 3 Centre for Environmental Policy, Imperial College London, London, UK; 4 Department of Zoology, University of Oxford, Oxford, UK; 5 Centers for Disease Control and Prevention (CDC), Atlanta, USA; 6 Centre for Applied Entomology and Parasitology, School of Life Sciences, Keele University, Staffordshire, UK; 7 Department of Life Sciences, Imperial College London, London, UK; 8 Laboratoire de Recherche et d’Enseignement en Santé et Biotechnologies Animales, Université Nazi Boni (UNB), Bobo-Dioulasso, Burkina Faso

**Keywords:** *Anopheles gambiae* (*s.l.*), Genetic control, Human settlements, Mosquito sampling outside villages, Vector control

## Abstract

**Background:**

One of the promising current approaches to curb malaria lies in genetic vector control, the implementation of which will require an improved understanding of the movement of genetic constructs among mosquito populations. To predict potential gene flow from one area to another, it is important to begin to understand mosquito dynamics outside of the commonly-sampled village areas, and thus how genes may move between villages. This study assessed the presence and relative abundance of mosquitoes in a 6-km corridor between two villages in western Burkina Faso.

**Methods:**

The area surrounding the villages was mapped and the road between them was used as the basis of a transect along which to sample. Five collection points were placed along this transect. To investigate both larval and adult mosquito presence, multiple sampling approaches were used surrounding each point: searching for larval sites in an area of 500 m radius, swarm sampling, human landing catches (HLC), CDC light traps and backpack aspiration catches of potential resting sites. Sampling took place twice: in September and October 2015.

**Results:**

Adult mosquitoes from six species of *Anopheles* and three other genera were found along the whole transect. *Anopheles gambiae* (*s.l.*) was the most abundant followed by *Anopheles nili* and *Anopheles coustani*. Larvae of *Anopheles* spp. were found in small pools of surface water along the whole transect, though their presence increased with human proximity. HLC and aspiration were the most efficient methods of collecting adult mosquitoes along the whole transect, indicating that there are both host-seeking and resting mosquitoes well away from core village areas. In contrast, swarms of male mosquitoes, thought to be the principle mating locations of *Anopheles* spp. mosquitoes in West Africa, were only found close to the core village areas.

**Conclusions:**

This preliminary study indicates that *Anopheles spp.* mosquitoes are both present and breeding in low human-density areas along transit axes and provides both a relative evaluation of methods for use in these areas and evidence that gene flow between Sahelian population centres is likely. More robust and structured studies are nevertheless needed to come with stronger conclusions.
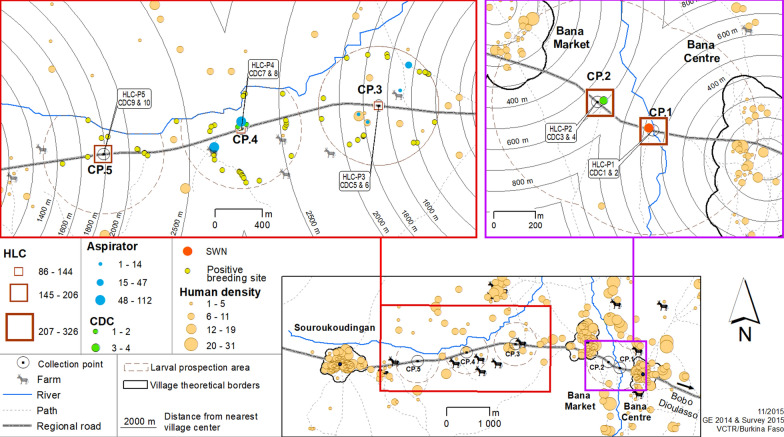

## Background

In a global context of increasing mosquito resistance to the main insecticide classes used in public health [1, 2], malaria control programmes in many sub-Saharan countries face reduced efficiency of their insecticide-based vector control tools. In consequence they observe a stabilisation of malaria incidence and morbidity [1, 3] and in some cases a resurgence of the disease [2, 4]. Substantial research efforts now focus on new vector control approaches or tools, such as genetic vector control [5–7], entomopathogenic bacteria or fungi [8, 9], and indoor mosquito traps [10, 11] to complement the strategies in current use.

Among the promising new vector control approaches, genetic tools to challenge malaria transmission *via* a reduction of vector density or vector capacity (such as increased vector resistance to *Plasmodium* parasite infection), have seen increasing interest this last decade. One of the major knowledge gaps associated with prediction, assessment or implementation of these tools is that of the gene flow potential surrounding intervention areas. Gene flow is predominantly intraspecific [12] and is essentially the product of mosquito movement from one place to another for blood-feeding, mating or larval development site-seeking, or because of environmental factors such as wind or human-borne [13]. As some of these proposed tools have potential for area-wide implementation, understanding gene flow *via* mosquito movement between neighbouring locations will be essential for predicting spread and cost-effectiveness.

Passive windborne movement of up to 300 km of *Anopheles coustani* has been reported [14, 15] and rare, long-distance, movements have been observed in *An. gambiae* [13, 16]. At smaller scale, and, in certain conditions these mosquitoes can fly up to 12 km [17, 18]; however, evidence suggests that most *Anopheles* mosquitoes do not move far in their lifetimes [19]. In Sahelian areas such as western Burkina Faso, in the dry season most villages are separated by extensive spaces considered hostile to mosquitoes because of low availability of resting sites (vegetation/houses) and the surface water necessary for larval development. In contrast, in the rainy season, human activities (agriculture, stock farming) extend out of villages into surrounding areas. Implementing a self-sustaining mating-mediated vector-control tool such as gene drive in such an ecological environment will be dependent on a sufficient level of intraspecific gene flow in the targeted area. Evidence of mosquito presence in these far less populated areas could be indicative of migration or continuous populations between villages and thus suggests a consequent level of gene flow. This evidence could also been indicative of potential support to local malaria transmission, as reserve of infection which could potentially hindered local malaria indoor-based vector control achievements (usually focussed within nearby significant human agglomerations). Monitoring mosquito presence and dynamics in the low human density areas between village agglomerations will bring important information to understand malaria vectors and transmission dynamic and thus to predict potential efficiency of genetic vector control tools.

In this initial study, the presence and abundance of mosquitoes in an area located between the villages of Bana and Souroukoudingan, two well-studied villages of western Burkina Faso [20–22], were assessed during the wet season, using several sampling methods. Designed as a preliminary study, the study aimed to evaluate methodology and provide initial estimates of mosquito abundance and diversity in support of future structured studies of malaria vector population dynamics and related gene flow in such zones. The use of several methods assessed both the relative efficiency of the methods and their potential for use outside the traditionally sampled village areas where pesticide spray catches (PSC) and sweep netting of swarms are most commonly used [22].

## Methods

### Study sites

**Fig. 1:**
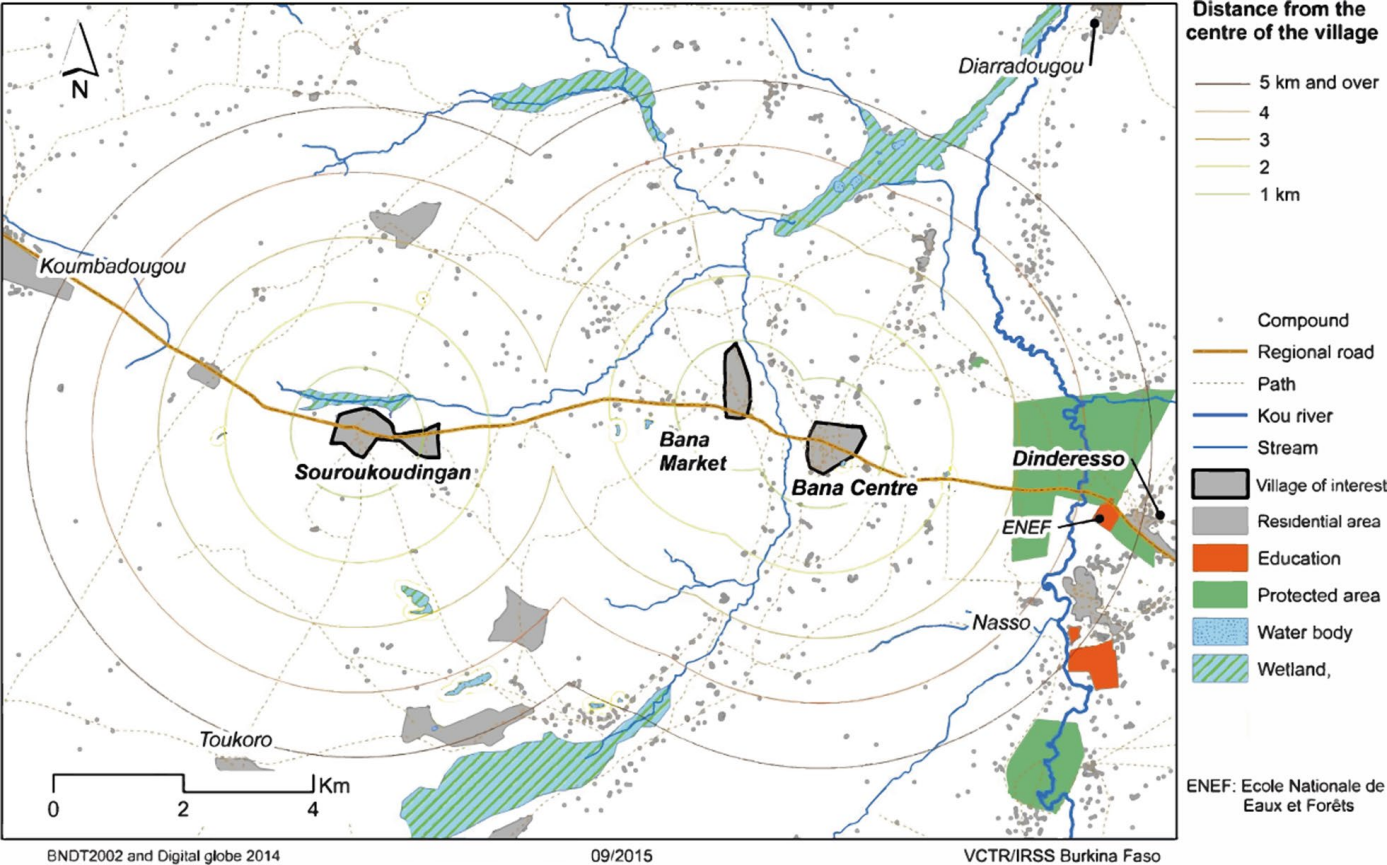
The study area and surrounding villages in western Burkina Faso

The survey was conducted between the villages of Bana and Souroukoudingan, two villages in western Burkina Faso’s humid savannah (Fig. 1). Bana is 23 km west of Bobo-Dioulasso (11°14′12″N, 4°28′40″W). It has two main human population agglomerations: Bana Centre (about 60 family compounds) and Bana Market (about 70 compounds), separated by 1.5 km. The village of Souroukoudingan (11°14′07″N, 4°32′11″W) is 6 km from Bana Market and their geographical and socio-demographic characteristics are similar. Souroukoudingan village is a single cluster of about 100 compounds.

### Entomological surveys

**Fig. 2:**
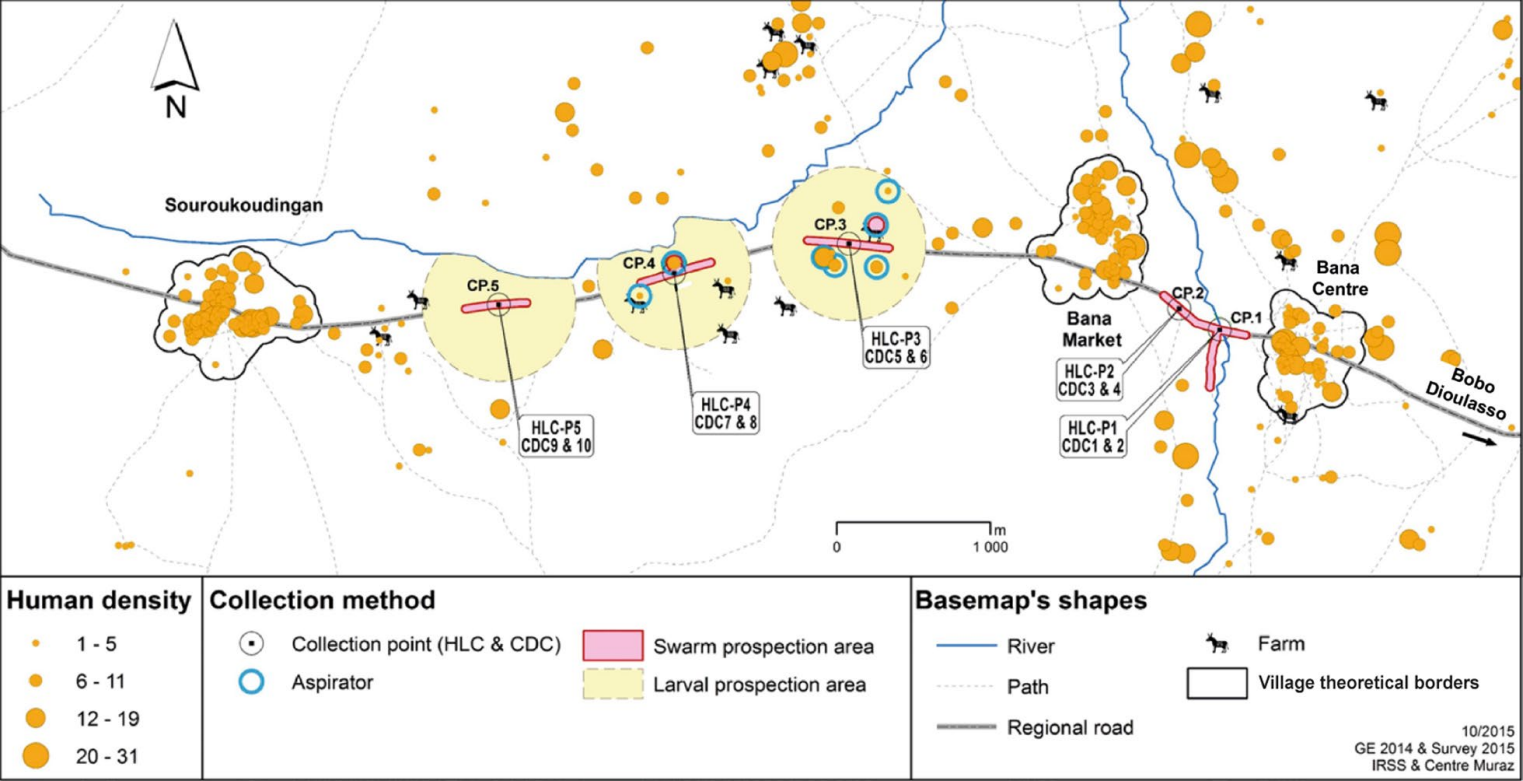
Entomological prospection methods. The areas in which entomological prospections were carried out (prospection areas) and the sampling methods used. *Abbreviations*: CP, collection point; HLC, human landing catches; CDC, CDC light trap sampling

Two similar entomological field surveys were carried out in September and October of the late 2015 wet season which includes the peak of mosquito abundance and the transition toward the start of dry season [22]. Five fixed collection points (CPs) were designated along the dirt road from Bana to Souroukoudingan. Two CPs were between Bana Centre and Bana Market and three between Bana Market and Souroukoudingan. These CPs were placed approximately equidistantly between the village borders (Fig. 2). The CPs were originally selected from satellite images of the area and their precise locations were determined after ground-truthing with specific criteria (accessibility and security for the field team). For seven days at each survey, a thorough mosquito prospection and collection was carried out in an area of 500 m in radius around each designated CP (the ‘prospection areas’). Five collection methods were used: larval habitat prospection, human landing catches (HLC), CDC light trap collection, backpack aspiration of potential resting sites and swarm collection using sweep nets.

#### Larval collections

A thorough survey of larval habitats was performed in the prospection areas using larval habitat search and sampling methods. During each survey, all water locations (potential larval habitats) were recorded, georeferenced using a GPS device (Garmin GPS series GPSMAP^®^62.2.3), and described physically (type, size). All were methodically scouted, according to the parent project protocol, for the presence of *Anopheles* larvae or pupae [22] which were identified morphologically in the field using the anopheline morphological identification keys developed by Holstein [23]. For each survey, larval habitat prospection and sampling were completed during two consecutive days. Sampling procedure aimed to detect productive larval habitats without further quantification or classification of immature mosquitoes collected. The low-dip technique was applied by a group of 8 well-trained individuals, using scoop-ladles (350 ml) and concentrator cups (fabricated locally). Finding at least one anopheline larva or pupa was sufficient to classify a particular larval habitat as ‘occupied’. No further quantitative estimates were made.

#### Adult collections

For two consecutive nights, two trained and supervised volunteers (from local communities), positioned at a distance of about 1 m from each other, collected outdoor, all human-attracted mosquitoes at each collection point from 20:00 to 01:00 h (five hours of collection). The choice of this particular collection period was driven by two main reasons: the security of collector (after discussion with local communities) and previous entomological studies in the main villages (Bana and Sourououdingan) which showed a mosquito host-seeking pattern with significant number of mosquitoes during that period of the night [22]. The same individuals were collecting mosquitoes at the same collecting point during the two days. At the same times, and at a minimum distance of 20 m from the volunteers, two standard CDC light traps (with light but no CO_2_ source), powered by a 12 V battery were also installed outdoor and hanged to distance of 1 m from the ground to collect mosquitoes.

Once, in daylight (07:00 to 10:00 h), at each survey, a backpack aspirator (CDC backpack, model 1412, John W Hock company, USA) was used indoor to search and collect mosquitoes in all identified potential resting places (agricultural huts, abandoned houses and livestock pens). Inside each settlement, aspiration was done during 5–10 min and targeted all available surfaces (ground, walls, roofs and eaves). At dusk, on two consecutive evenings, a team of experienced swarm samplers explored a 500 m roadside transect and used sweep nets to capture swarming mosquitoes.

#### Mosquito identification

Adult mosquitoes were identified morphologically in the field using Holstein’s adult anopheline morphological identification keys [23] and a field stereomicroscope (Perfex Sciences^®^ Zoom Pro. Reference: S0852Z5, Toulouse, France). Only adult *Anopheles gambiae* (*s.l.*) were further identified to sex and preserved in 80% (v/v) ethanol. The other *Anopheles* spp. and mosquitoes from other genera were just identified morphologically, counted and recorded.

### Data analysis

Mosquito abundance within each genus considered here was analysed as a function of survey month, collection point and method used. The exception to this is for the six component species of ‘other *Anopheles* spp.’ where the numbers collected were too low to reliably consider effects of method and collection point, and only the variation between survey was analysed statistically. Mosquito numbers (excepted for *An. gambiae* (*s.l.*)) refer to the total of both male and female. Parameters were estimated and compared using either proportion tests or binomial-family generalized linear models (GLMs) appropriate to the overdispersion found in these data. All statistical analyses were performed using R 3.3.1 [24].

A logistic regression was additionally used to assess whether larval presence was spatially associated to human proximity. For each larval site at location $x$, “Human proximity”, $H\left( x \right)$, was calculated as the number of human-occupied compounds within a distance of 500 m from $x$. Larval site observations were excluded from the regression if they were repeat observations at the same site to give the same result, or if they were from a site where repeated observations gave different results. This yielded 90 observations (55 presences and 35 absences), whose locations and human proximities are shown in Additional file 1: Figure S1.

## Results

**Fig. 3:**
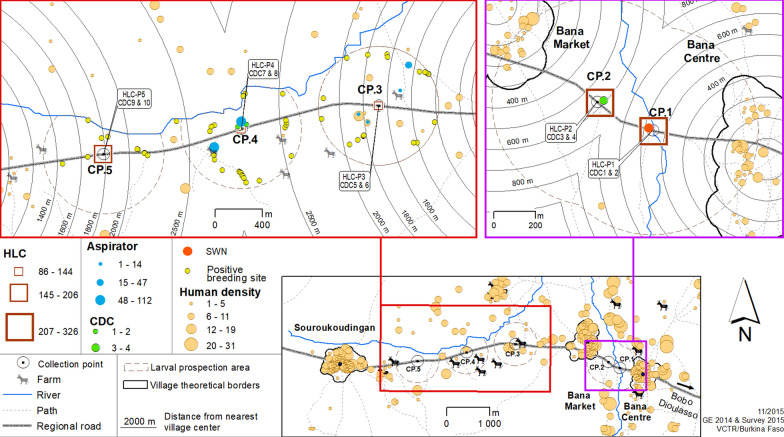
*Anopheles gambiae* (*s.l.*) abundance in collections between the villages of Bana and Souroukoudingan during the September 2015 survey. Numbers of mosquitoes collected by sampling method and collection point (CP). *Abbreviations*: Aspirator, backpack aspirator sampling; HLC, human landing catches; CDC, CDC light trap sampling, SWN, sweep netting of swarm sampling

**Fig. 4:**
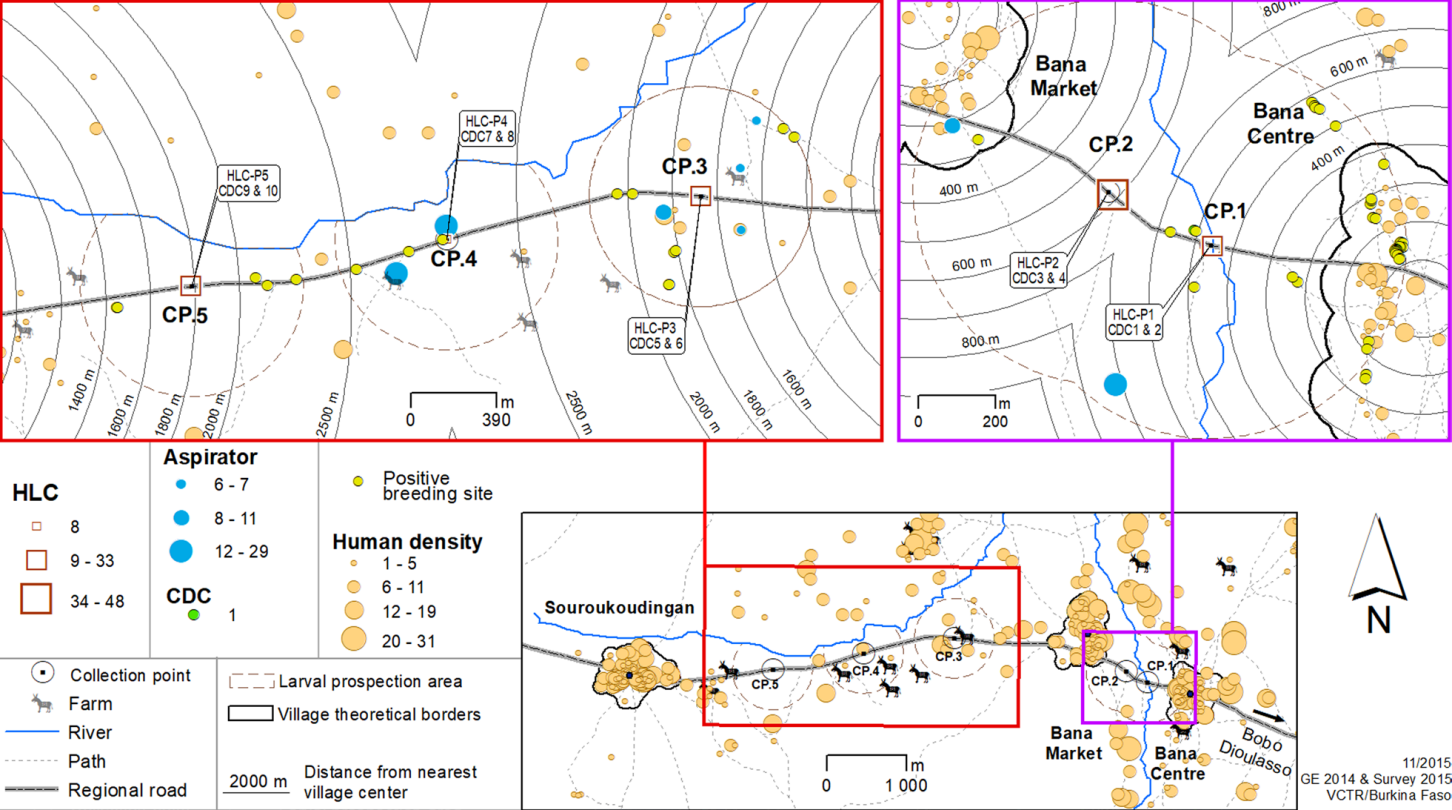
*Anopheles gambiae* (*s.l.*) abundance in collections between the villages of Bana and Souroukoudingan during the October 2015 survey. Numbers of mosquitoes collected by sampling method and collection point (CP). *Abbreviations*: Aspirator, backpack aspirator sampling; HLC, human landing catches; CDC, CDC light trap sampling; SWN, sweep netting of swarm sampling

Both the September 2015 (Fig. 3) and October 2015 surveys (Fig. 4) found appreciable numbers of mosquitoes along the transect between Bana to Souroukoudingan despite the scarcity of nearby human habitations.

### Larval habitat surveys

**Fig. 5:**
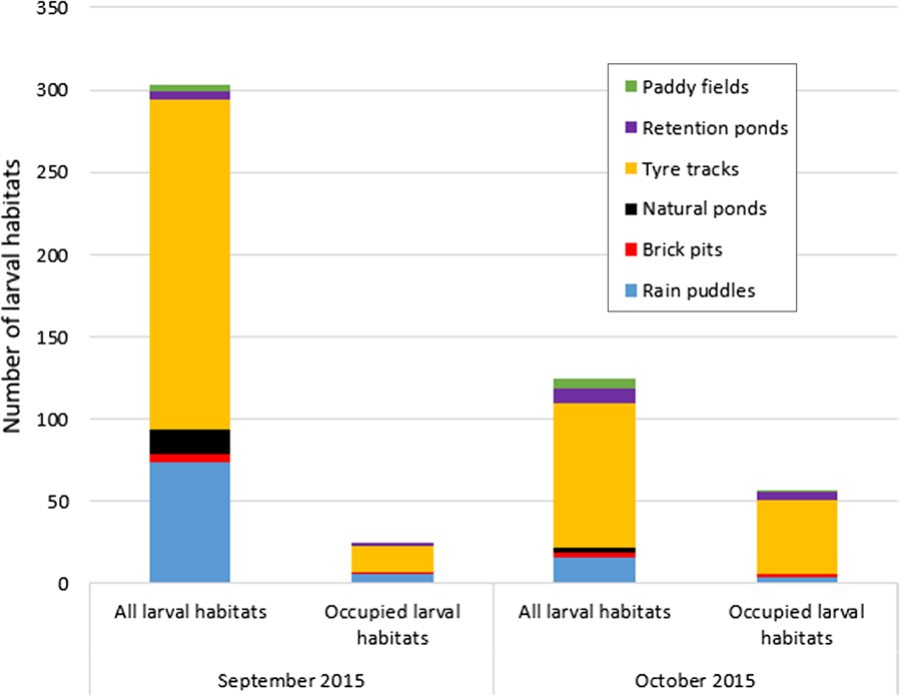
The types and abundance of potential and occupied larval habitats found in the prospection areas in each survey period

Prospection of larval habitats in between the village of Bana and Souroukoudingan identified 303 potential larval sites in September 2015 and 125 in October 2015. A lower proportion of the sites was identified as being occupied by *Anopheles* mosquitoes in September (8.25%, 25/303) than in October (45.6%, 57/125) (*χ*^2^ = 77.31, *df* = 1, *P* ˂ 0.001). The majority of these larval sites consisted of tyre tracks and puddles (Fig. 5) which are short term and highly rain-dependent [22]. Overall, these two ephemeral larval site types represented 90% of all available larval sites in September and 83% in October 2015. About 88% and 86% of the occupied larval sites found in September and October 2015, respectively, were also from these two types.

**Fig. 6:**
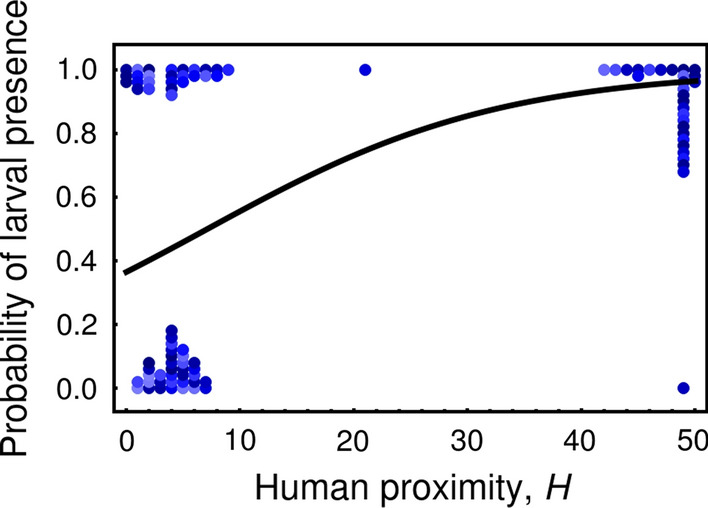
Logistic regression estimating the probability of occupation of a larval habitat as a function of ‘H(x)’, the number of human-occupied compounds within 500 m from $x$. The regression curve is given by the equation $prob = \frac{1}{{1 + e^{a - b H\left( x \right)} }}$, where $a = 0.55$ and $b = 0.078$

The logistic regression indicated a positive relationship between human proximity and the presence of *An. gambiae* larvae; there was greater site occupancy near human-occupied compounds (Fig. 6). This regression, however, also suggests that isolation at the scale of 100 m is not a barrier to oviposition; the most isolated sites examined in this study were occupied with probability of about 0.37.

### Adult mosquito surveys

#### *Anopheles gambiae* (*s.l.*)

The number of *An. gambiae* (*s.l.*) (total of both male and female) captured varied with both month (*F*_(1, 35)_ = 33.15, *P* < 0.001) and the method used (*F*_(3, 35)_ = 28.95, *P* < 0.001), though not as a function of the collection point (*F*_(4, 31)_ = 0.49, *P* = 0.74) (Figs. 3, 4; Table 1). HLC (female-specific sampling method) caught the most mosquitoes (male and female included), followed by aspiration of resting shelters, while the CDC light traps (female-specific sampling method) and swarm sampling (male-specific sampling method) caught only small numbers.

**Table 1:** The number of adult mosquitoes collected in total and within each prospection area as a function of the method used

Area	Method	*An. gambiae* (*s.l.*)		Other *Anopheles*		*Culex* spp.		*Aedes* spp.		*Mansonia* spp.	
		Sep	Oct	Sep	Oct	Sep	Oct	Sep	Oct	Sep	Oct
CP 1	HLC^a^	326	25	78	15	3	5	75	9	20	34
	CDC^a^	4	1	0	2	0	2	0	1	0	0
	SWN^b^	17	0	0	0	0	0	10	0	0	0
	ASP	0	20	0	1	0	1	0	0	0	0
			(5; 15)								
CP 2	HLC^a^	280	44	36	10	4	0	25	19	16	62
	CDC^a^	0	0	1	0	1	2	1	0	2	1
	SWN^b^	0	0	0	0	0	0	0	0	0	0
	ASP	0	9	0	0	0	0	0	0	0	0
			(8;1)								
CP 3	HLC^a^	144	18	22	11	5	3	50	28	1	28
	CDC^a^	0	0	0	0	8	0	0	0	0	0
	SWN^b^	0	0	0	0	0	0	0	0	0	0
	ASP	73	30	0	1	0	0	0	0	0	0
		(37; 36)	(19; 11)								
CP 4	HLC^a^	86	8	28	1	1	2	40	112	0	1
	CDC^a^	3	0	0	0	2	0	0	1	0	0
	SWN^b^	0	0	0	0	0	0	0	0	0	0
	ASP	199	40	3	20	8	3	0	0	0	0
		(123; 76)	(12; 28)								
CP 5	HLC^a^	206	25	30	14	4	1	58	19	17	7
	CDC^a^	0	0	0	0	0	0	0	0	0	0
	SWN^b^	0	0	0	0	0	0	0	0	0	0
	ASP	0	0	0	0	0	0	0	0	0	0
Total	HLC^a^	1,042	120	194	51	17	11	248	187	54	132
	CDC^a^	7	1	1	2	11	4	1	2	2	1
	SWN^b^	17	0	0	0	0	0	10	0	0	0
	ASP	272	120	3	22	8	4	0	0	0	0
		(160; 112)	(44; 55)								

^a^Female-specific sampling method (numbers represent only female mosquitoes)

^b^Male-specific sampling method (numbers represent only male mosquitoes)

*Notes*: Numbers in parentheses indicate respectively the specific number of female and male mosquitoes caught. Mosquitoes other than *Anopheles gambiae* (*s.l.*) were not recorded by sex (so the numbers indicated represent addition of both male and female caught)

*Abbreviations*: Sep, September 2015; Oct, October 2015; CP, collection point; ASP, aspiration sampling of mosquito resting sites with backpack aspirator; HLC, human landing catches; CDC, CDC light trap sampling; SWN, sweep netting sampling of swarms

Male *An. gambiae* (*s.l.*) were found in backpack aspirator and swarm samples. Their relative proportion varied between the September and October surveys (*χ*^2^ = 33.19, *df* = 1, *P* ˂ 0.001). In September, 129 males of *An. gambiae* (*s.l.*) mosquitoes were captured (17 in two swarms and 112 by backpack aspirator), representing about 10% of all *An. gambiae* (*s.l.*) collected. In October, this proportion was higher at about 23% (55 male mosquitoes all collected by backpack aspirator).

#### Other *Anopheles* species

Other anophelines were fewer in number and followed a similar pattern with more caught in September (*F*_(1, 35)_ = 5.51, *P* = 0.025), more caught by HLC than any other method (*F*_(3, 35)_ = 17.81, *P* < 0.001) and, when pooled in this way, there was no identifiable variation in catch along the transect (*F*_(4, 31)_ = 0.92, *P* = 0.47). However, the data hint that there may be species-specific differences in temporal trend (Table 2). The malaria vector *An. nili* declined substantially from September to October in a similar manner to *An. gambiae* (*s.l.*), yet *An. coustani* and *An. pharoensis* did not display any identifiable temporal variation. All other species identified, including the major malaria vector *An. funestus*, rose in proportion (Table 2). The low numbers of these rarer species were not sufficient to analyse the relative efficiencies of the capture methods.

**Table 2:** Percent abundance of each adult ancillary *Anopheles* spp. mosquito collected in 2015 as a function of survey month

*Anopheles* spp.	September (*N* = 198)	October (*N* = 75)	*χ*^2^	*df*	*P*
*An. coustani*	25%	27%	0.03	1	0.86
*An. flavicosta*	–	23%	44.06	1	< 0.001
*An. funestus* (*s.l.*)	5%	29%	28.69	1	< 0.001
*An. nili*	68%	9%	71.83	1	< 0.001
*An. pharoensis*	1%	1%	0.00	1	0.99
*An. rufipes*	1%	11%	11.77	1	< 0.001

Statistics estimate whether these proportions vary between collection months

*Abbreviations*: *N*, the total number of mosquitoes caught during the given period

#### Other genera

There was no evidence that *Culex* spp. mosquitoes varied in number along the transect (*F*_(4, 31)_ = 1.29, *P* = 0.30), nor was there evidence of a different catch level between the months (*F*_(1, 35)_ = 2.36, *P* = 0.13). There was some variation as a function of the method used; this was largely driven by the absence of any *Culex* spp. found in swarms (anopheline-oriented collection method); all other methods performed indistinguishably with these low capture numbers (*F*_(3, 36)_ = 4.90, *P* = 0.006). *Aedes* spp. mosquito numbers were similar in the two months (*F*_(1, 31)_ = 1.82, *P* = 0.19), but were seen to vary in number along the transect (*F*_(4, 31)_ = 2.81, *P* = 0.042) and by method (*F*_(3, 32)_ = 58.15, *P* < 0.001) as the HLC at CP4, the most distant from either village, was particularly productive. *Mansonia* spp. displayed a different pattern and numbers varied along the transect (*F*_(3, 32)_ = 13.81, *P* < 0.001) with the majority being caught at CPs 1 and 2 near the river between the Bana agglomerations. Unlike the other mosquitoes, more of this genus were caught in October than September (*F*_(1, 31)_ = 23.22, *P* < 0.001) and the method used also mattered: HLC caught all but 3 of the 190 individuals (*F*_(3, 31)_ = 118.17, *P* < 0.001) (Table 1).

## Discussion

The identification of a diversity of mosquito species all along a transect between villages indicates that, in this Sahelian region, significant populations exist outside of the typically-sampled inhabited areas. The choice of the linking road as the basis for a transect was in part pragmatic (safety and practicality of sampling) and an acknowledgment that anthropophilic mosquitoes, if any present, are likely to be found along human transport routes. The three principal malaria vectors of Burkina Faso: *An. gambiae* (*s.l.*), *An. funestus* (*s.l.*) and *An. nili* [25, 26] were all found, along with species from three other genera. Although *An. gambiae* (*s.l.*) dominated the catch, the relative proportions of *An. nili* and *An. funestus* (*s.l.*) were unexpectedly high when compared to the proportion observed in the villages of Bana and Souroukoudingan during the same period [22]. This pattern may result from the intense competition for food and larval habitat sites between the vector species in each-others’ presence [27–29] or the fact that they are less strongly anthropophilic. The seasonal dominance of *An. gambiae* (*s.l.*) mosquitoes may drive *An. nili* and *An. funestus* (*s.l.*) to areas more distant from core human habitat which may thus form refuges from interspecific competition during larval stages [28]. The types of larval habitat generally found in these areas are not generally considered favourable to the development of *An. nili* and *An. funestus* (*s.l.*) larvae, which are known to prefer running water with rapid flow or stagnant water with submerged vegetation, respectively [30, 31]. With this in mind, it may just be that adult dispersal of these species is widely underestimated.

Comparison of the sampling methods used underlines the difficulty in finding a reliable (efficient and easy to implement) collection method to detect and monitor mosquito populations in areas outside villages. This is largely driven by the fact that these methods showed differential easiness in implementation and target different groups or types of mosquitoes (male, female or both male and female, host-seeking or resting mosquitoes). Overall, though it caught less than the HLC, aspiration of potential resting places did deliver a large number of *Anopheles* spp. mosquitoes of both sexes and some *Culex* spp., but found no *Aedes* spp. nor *Mansonia* spp. Aspiration is practical and can be carried out during the day, but has limitations when potential resting places are scarce such as in open spaces with little vegetation. Human landing catches were effective for capturing host-seeking females of all genera seen here (female-specific sampling method), but must take place overnight, require substantial human effort, raise ethical questions and are impractical in remote locations. Very few female mosquitoes were caught *via* CDC light traps compared to adjacent HLC sampling. These fewer catches may be explained by the lack of a stimulant (CO_2_) as the standard setting of this trap usually use a human person under a bed-net as a bait [32]. It is also known that this trap yields fewer catches when used outdoors [33]. The relative proximity with HLC sampling in this study may also have had a negative influence on CDC light trap performance. Swarm sampling (male-specific sampling method) is very efficient for male *Anopheles* spp. mosquito monitoring inside villages [21, 34, 35]. Very few swarms were found in between villages though the sampling team has substantial experience in this technique, and the majority of male mosquitoes were collected through backpack aspirator sampling. As observed in previous studies, male mosquito swarming appears to be closely associated with the presence of human habitation [36].

New adult *Anopheles* outdoor collection methods are being developed and optimised. Some of these could be explored to provide monitoring of mosquito populations in uninhabited areas. Some of the most promising are the Biogent sentinel traps [37], Mosquito Magnet traps [38, 39], improved CDC light traps [40], improved mosquito trapping boxes [41], and the Suna trap [42]. These methods, however, do have various requirements in terms of power supply and regular servicing though several have the advantage of some autonomy, mobility, and are cost-effective. Mosquito electrocuting traps [43] have also been proposed but incur some of the same limitations as HLC in remote areas. This is also the case of other exposure-free sampling tools such as the human baited double net trap (HDN) [44, 45] or the host decoy trap (HDT) [46]. In fact, the special configuration of such areas (low accessibility, vegetation coverage, presence of potentially dangerous wild animals) renders difficult, well designed implementation of human baited sampling techniques. These trap types also vary in their ability to provide living mosquitoes when these are needed for purposes including bioassays and colony establishment.

Though larval habitat occupancy is lower than in the adjacent villages [22], there is also much promise in the use of larval habitat monitoring. *Anopheles* spp. larvae were found in several types of ephemeral and opportunistically available habitats, and this does suggest that searching for and sampling these can be rewarding, most especially in terms of presence/absence of a species or genotype. The presence of such immature stages of *Anopheles* mosquitoes suggest the presence of adult mosquitoes around, either from a local population or from nearby villages migration. The high level of larval habitat occupancy seen in the October sample, when background numbers of sites had fallen as the rainfall declined, raises a further possibility not used in this study, but which may prove practical for these out-of-village settings; the use of simple ovitraps. These are unlikely to provide well-calibrated quantitative estimates due to seasonal variation in relative attractiveness in comparison with natural settings, but might also prove useful as a presence/absence indicator complement in seasons when naturally occurring larval sites are rare. They have been widely used in other settings [47–49] and do have advantages of low-cost, easy distribution and low risks. The water they contain has, however, been noted to attract a wide range of thirsty animals (NJ Besansky, pers. comm.).

The dominant paradigm for West Africa is that swarms are the principal mating locations for *Anopheles* spp. [34, 50, 51], with the paucity of swarms found along the transect, it is certainly plausible that their ecology varies away from high density inhabited settings and that copulation in these areas is more opportunistic. This has long been thought to be the case in East Africa where swarms are also rare. Larval presence and the numbers of mosquitoes caught by HLC while seeking blood meals indicates that mating must happen in these areas and may be promoted by human transit along axes between villages. Here, pedestrian transit and vehicles such as cars, trucks and bicycles compact the soil and promote surface pooling. People, along with their agricultural animals, also provide opportunistic blood meals. Although the mosquito populations are likely to be more fragmented during dry seasons, displaying a seasonally reticulated pattern, the high probability of larval site occupancy at substantial distances from human habitations warrants further exploration. There is an enormous amount to discover about *Anopheles* vector ecology in low-human density areas and further study outside villages to give a better understanding of the structure of these mosquito populations would be rewarding.

The substantial fall in mosquito and larval habitat numbers from September to October indicates a highly seasonally-mediated dynamic in such settings. In September (mid-wet season) mosquitoes are found in some numbers outside villages leading to possible gene flow between neighboring areas. When the dry season approaches, as observed here in October (typically a wet-dry transitional month in this region), this out-of-village abundance declines and suggests the possibility of complete interruption in mosquito migration in the core dry season. This study goes some way towards addressing a long standing contradiction that has been noted between apparent high gene flow in *An. gambiae* (*s.l.*) in West Africa [52] and the paradigm that *An. gambiae* (*s.l.*) mosquitoes are only found in close proximity to human habitation [34, 50]. Longer-term (full year) monitoring and a more structured empirical data collection could be of great interest in understanding this potentially pulsating pattern of population connection and the consequent population reticulation it implies.

Successful implementation and cost-effective deployment of future genetic vector control tools will rely on the ability of the gene of interest to move from one place to another. Thus, understanding potential gene flow through mosquito movement between villages and through low human density areas becomes crucially important. In many rural Sahelian areas, villages may be distant from each other with intervening areas usually viewed as unsuitable to mosquito development. This study has found evidence of mosquito presence and breeding during the wet season in an almost uninhabited, but roadside, area in between two neighbouring villages of western Burkina Faso. It is likely that larval habitat availability and the humidity associated with wet seasons facilitates a pulse of background mosquito populations into mostly inhospitable areas and that this in turn creates a seasonal connectivity that can support gene flow along transport axes. Although both study design and results from this study are not enough to confirm the existence of gene flow between the villages, they do demonstrate that substantial investment in a better understanding of mosquito seasonal and spatial dynamics in such settlements are worthy of consideration. Further studies are needed to evaluate the presence and abundance of mosquitoes away from transport axes and this is contingent on the use of practical sampling techniques.

## Conclusions

An important and diverse mosquito fauna including malaria vector species was found in the area between the villages of Bana and Souroukoudingan. This may be indicative of mosquito migration from one village to the other and thus the possibility of intraspecific gene flow between mosquito populations from neighbouring villages in this Sahelian region. To confirm this hypothesis, more structured studies (consistent sampling effort and empirical data collections) will be needed to properly assess mosquito migration in such settlements. To improve our understanding of these mosquito populations, appropriate mosquito collection tools should also be used or developed having in mind ease of use and deployment, cost effectiveness and efficacy.

## Supplementary information

**Additional file 1: Figure S1.** Logistic regression model of human proximity to observed larval sites in areas between Bana and Souroukoudingan villages. Map of compounds and larval sites (top) used in the logistic regression model. The positive (middle) and negative (bottom) larval sites are plotted as circles with area relative to the number of compounds within 500 m of their location (‛human proximityʼ, H).

## Data Availability

The datasets generated and/or analysed during the present study are not publicly available due to the fact that they are part of a larger research project that is still ongoing; but are available from the corresponding author on reasonable request.

## Abbreviations

GLM: generalized linear models
GPS: global positioning system
CDC: Centers for Disease Control and Prevention
HLC: human landing catches
PSC: pesticides spray catch
v/v: volume for volume
