# Supplementary figures and images for: Anopheles gambiae (s.l.) is found where few are looking: assessing mosquito diversity and density outside inhabited areas using diverse sampling methods
Source: Parasit Vectors. 2020 Oct 15;13:516. doi: 10.1186/s13071-020-04403-9 (PMC7558606; doi:10.1186/s13071-020-04403-9)

- positive larval site    ● negative larval site
- inhabited compound

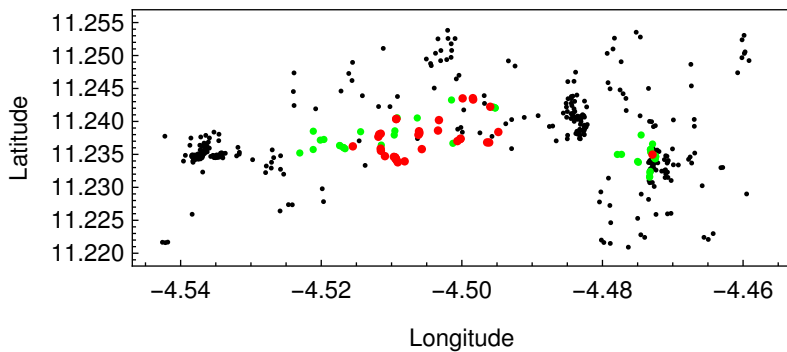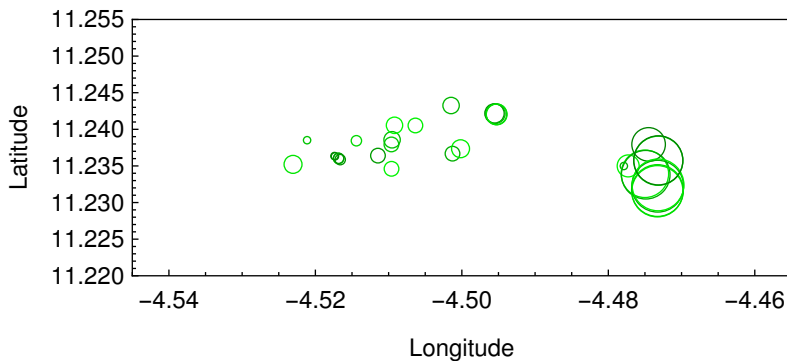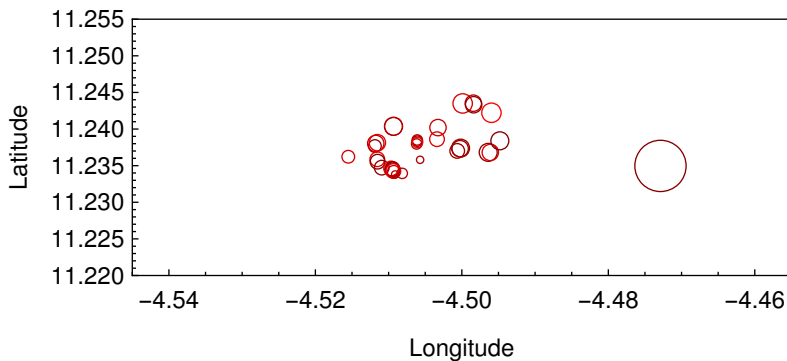

Supplement: Supplementary file 1 — Additional file 1: Figure S1. Logistic regression model of human proximity to observed larval sites in areas between Bana and Souroukoudingan villages. Map of compounds and larval sites (top) used in the logistic regression model. The positive (middle) and negative (bottom) larval sites are plotted as circles with area relative to the number of compounds within 500 m of their location (‛human proximityʼ, H). [file 13071_2020_4403_MOESM1_ESM.pdf]
